# Synchronous polypectomy during endoscopic diagnosis of colorectal cancer – is the risk of tumour implantation at the polypectomy site significant?

**DOI:** 10.1186/s12876-018-0861-4

**Published:** 2018-08-29

**Authors:** W. J. Tan, N. ZP. Ng, Y. D. Chen, Y. H. M. Chee, F. J. Foo, C. L. Tang, M. H. Chew

**Affiliations:** 1 Department of Colorectal Surgery, Singapore General Hospital, 20 College Road, Academia Level 5, Singapore, 169856 Singapore; 2 Department of General Surgery, Sengkang General Hospital, 110 Sengkang East Way, Singapore, 544886 Singapore

**Keywords:** Colorectal cancer, Synchronous polypectomy, Tumour seeding, Local recurrence, Metachronous tumour

## Abstract

**Background:**

Synchronous polypectomy in colonic malignancies is contentious due to the perceived risks of tumour implantation at polypectomy sites (PS). We assess the risks of tumour implantation after synchronous polypectomy.

**Methods:**

An analysis of all endoscopies for cancer that were accompanied by synchronous polypectomies from 2005 to 2009 was performed. The incidence of metachronous colorectal cancers located at the same segment of a previous PS was the surrogate for tumour implantation. Data on patient demographics, tumour and polyp location(s) and follow-up outcomes were extracted. The rate of metachronous lesions at the same segment of a previous PS between patients who had all synchronous PS resected (Group A) and patients with PS left in-situ (Group B) were compared.

**Results:**

Two hundred and eighty-four patients had synchronous polypectomy performed during their initial endoscopy for cancer. Three patients were lost to follow-up and, in the remaining 281 patients, 87 (31.0%) were in Group A while 194 (69%) were in Group B. Median age, gender, tumour location, tumour stage, and pathological characteristics were similar between both groups. 2 (0.7%) patients developed local recurrences. Six (2.1%) patients developed metachronous lesions, four of which were located at the same segment where synchronous polypectomy was previously performed. The rates of metachronous lesions at the PS in groups A and B were similar at 1.1% (1/87) and 1.5% (3/194), respectively (*p* = 0.795).

**Conclusion:**

Malignant implantation after synchronous polypectomy in the setting of a newly diagnosed cancer remains unproven. Even if tumor implantation did occur, the incidence is likely low.

## Background

Colonoscopy is an essential component of colorectal cancer diagnosis. Synchronous polyps are detected in up to 15–50% of malignancies during colonoscopy [1–4].

As viable cancer cells exfoliated into the lumen have a recognized role in anastomotic suture line implantation and tumour recurrence with the normal physiological passage of faeces [5–8], many endoscopists prefer not to perform immediate polypectomy of synchronous polyps due to a perceived risk of tumour cell implantation into the raw colonic mucosa left at the polypectomy site. It is uncertain whether doing so protects patients from a theoretical and unquantified risk of tumour implantation. In turn, such an approach subjects patients to a further endoscopic procedure that could have been avoided had a polypectomy been performed during the initial diagnostic colonoscopy. This formed the premise of an earlier systemic review by Sheel et al. [9]. In this review, articles were included if they described human or mammalian models related to colorectal cancer cell implantation on normal and damaged colonic mucosa, such as polypectomy sites. The systematic review failed to identify any randomized trials, cohort studies, or retrospective evidence that addressed this clinical question. Available evidence was restricted to that of isolated case reports, the majority of which described implantation on haemorrhoidectomy or fistulectomy wounds. There was only a single case that described a possible cancer cell implantation into an endoscopic biopsy site proximal to a tumour [10].

The scarcity of evidence on this important clinical question prompted a review of our institutional data to decipher if any trends could be derived to guide future management. We decided to use the incidence of metachronous colorectal cancers located at the same colonic segment of previous polypectomy sites (PS) as a surrogate measure for possible tumour implantation. This study aims to compare the incidence of metachronous colorectal cancers in patients with resected PS to those whose PS were left in-situ following their primary colonic resection.

## Methods

An analysis of a prospectively maintained database of all patients who underwent curative resection for colorectal cancers from 2005 to 2009 in the Department of Colorectal Surgery at Singapore General Hospital was performed. All patients whose initial diagnostic endoscopy was accompanied by a synchronous polypectomy were included in our study. The study duration was chosen as it ensured that all patients included in the study had at least a 5-year duration of follow-up.

Patients who presented with recurrent cancer, inflammatory bowel disease, familial adenomatous polyposis, or other polyposis syndromes were excluded. We also excluded cases that were diagnosed on presentation with a stage 4 disease and those who underwent an endoscopic removal of malignant colorectal polyps or a local/transanal excision of low rectal cancers. The study protocol was approved by the Institutional Review Board of Singapore General Hospital.

Data on patient demographics, tumour and polyp location(s), biopsy method, size, morphology, histology, and follow-up outcomes were extracted. Patients who developed metachronous lesions during their follow-up were identified and details were sought to determine if these lesions were located at the same colonic region where their previous synchronous polypectomy was performed.

Patients were followed-up to the end of 2016 or to the day of their demise.

The cohort was divided into two groups for statistical analysis. Group A comprised patients who had all synchronous PS resected during their colorectal resection while Group B comprised patients with PS left in-situ.

### Follow-up details

The follow-up regime in our department is in accordance with the National Comprehensive Cancer Network guidelines [11]. At each consultation, carcinoembryonic antigen (CEA) levels were measured, and full history and physical examinations (including digital rectal examination) were performed. Patients are followed-up at thrice-monthly intervals for the first 2 years, bi-yearly for the next 3 years, then yearly thereafter. Colonoscopy was performed within 6 months of surgery for patients who did not have a complete colonic evaluation prior to their resection. Those who had an initial complete colonic evaluation would undergo colonoscopy at the first year of follow-up and again at 3-yearly intervals post-operatively if there were no indications for more frequent surveillance. Patients with suspicious symptoms and signs of rising CEA trend on follow-up would be evaluated earlier with colonoscopy and/or radiological imaging (including computerized tomography of the chest, abdomen, and pelvis, bone scan, and positron emission tomography scans if applicable).

### Adjuvant therapy regime

Adjuvant therapy was offered for all stage 3 patients who were deemed fit enough to undergo adjuvant chemotherapy. Adjuvant chemotherapy was also offered to stage 2 patients with high risk factors such as perineural invasion, lympho-vascular invasion, and obstructed or perforated tumours. Adjuvant therapy comprised 6 months of fluoropyrimidine (5-fluorouracil or capecitabine) with or without oxaliplatin.

We used metachronous lesions developing at the same colonic segment of the previous polypectomy as a surrogate measure for the possible risk of tumour seeding on PS.

Metachronous colorectal cancer was defined as a secondary colorectal cancer that occurred for more than 6 months after a curative resection of the index cancer [12]. Metachronous lesions developing near the anastomosis were differentiated from local recurrences by using 3 cm as a cut-off. Lesions at or within 3 cm of the anastomosis were considered local recurrences rather than metachronous lesions.

The location of the metachronous tumours detected on surveillance was determined based on their described location in the endoscopy report and computed tomography scan report. In all these lesions, details of the initial endoscopy with synchronous polypectomy were retrieved to determine if the lesion was located at the same colonic region where the synchronous polypectomy was performed.

### Statistical analysis

Differences between groups A and B were analysed using Fisher’s exact test for categorical variables while Mann-Whitney U test was utilized for continuous variables. Metachronous cancer rates at the colonic segments of previous synchronous polypectomy sites were compared between the two groups.

All statistical analyses were performed using SPSS version 20.

## Results

**Fig. 1:**
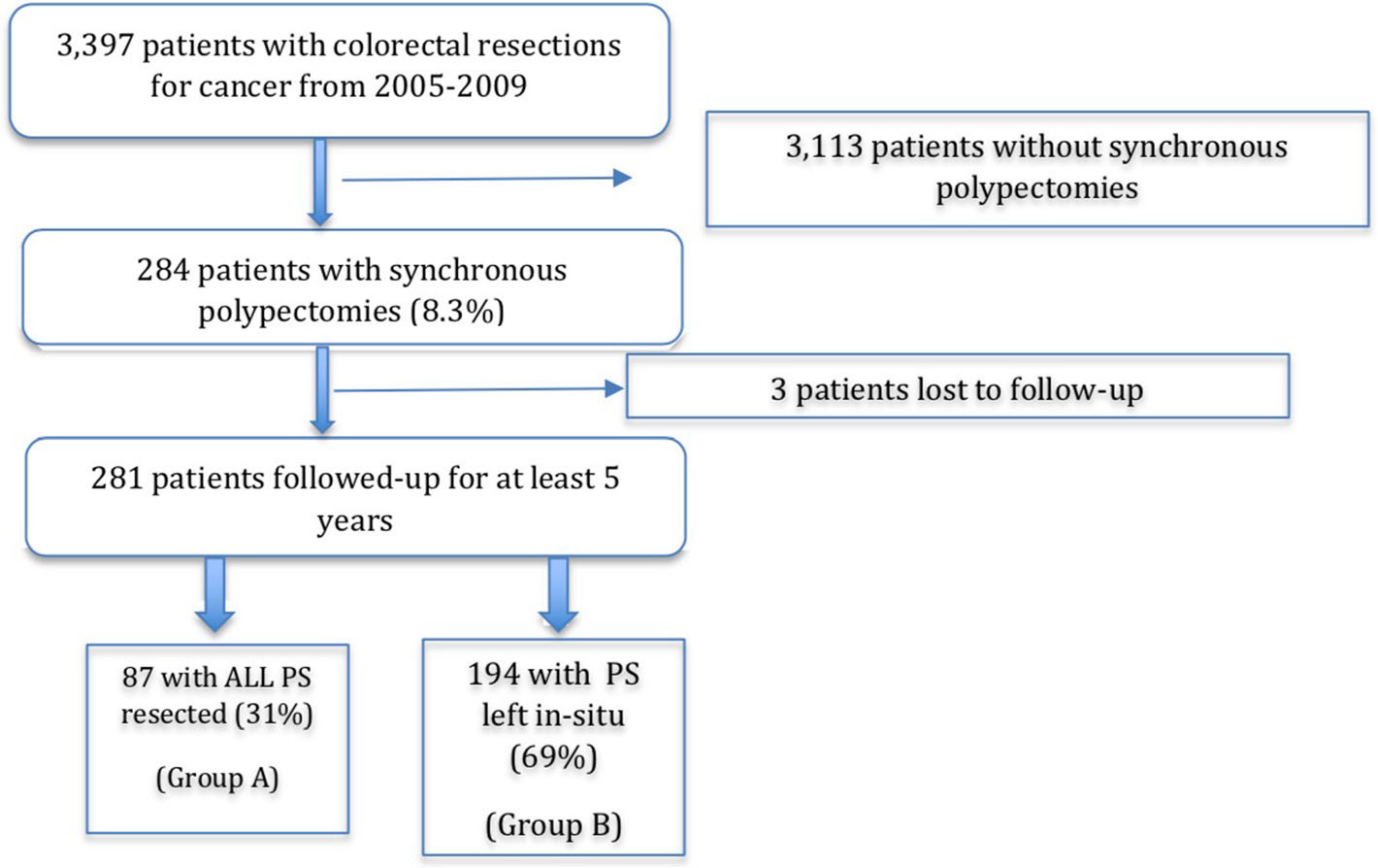
Flow diagram illustrating patient distribution of study cohort

Three thousand, three hundred, and ninety-seven patients underwent surgical resection for colorectal cancer from 2005 to 2009. Two hundred and eighty-four (8.3%) patients had polypectomy(s) performed for synchronous benign polyps at their initial diagnostic colonoscopy. Three patients were subsequently lost to follow-up and, among the remaining 281 patients, 87 (31.0%) had all their PS resected (Group A) while 194 (69%) patients had some or all PS left in-situ (Group B). The patient distribution of the study cohort is illustrated in Fig. 1.

A total of 469 polyps were removed in these 284 patients. The histological distribution of these polyps is illustrated in Table 1.

**Table 1:** Histology distribution of resected polyps

Polyp histology	Group A*	Group B^	Total Number (%) *n* = 469
Hyperplastic	31	88	119 (25.4)
Serrated adenoma	13	9	22 (4.7)
Tubular adenoma	77	207	284 (60.6)
Tubullovillous adenoma	11	24	35 (7.5)
Villous adenoma	0	1	1 (0.2)
Data missing	3	5	8 (1.7)

Group A* - Patients who had all synchronous polypectomy sites resected

*Group B^ - Patients who had synchronous polypectomy sites left* in situ

The demographic characteristics of the patients in groups A and B are illustrated in Table 2. Median age (67 years versus 70 years, *p* = 0.25) and gender distribution (70.1% males versus 63.9% males, *p* = 0.348) were comparable between groups A and B. Both groups did not differ significantly in terms of age, gender, tumour sites, T and N staging, or lympho-vascular and peri-neural invasion.

**Table 2:** Comparison of demographic characteristics between Groups A* and B^

	Group A * (*n* = 87)	Group B^ (*n* = 194)	*p*-value
Median age (range)	67 (34–92)	70 (32–94)	0.25
Gender			0.348
Male (%)	61 (70.1%)	124 (63.9%)	
Female (%)	26 (29.9%)	70 (36.1%)	
Tumor site, n (%)			0.838
Caecum	4 (4.6%)	13 (6.7%)	
Ascending	5 (5.7%)	21 (10.8)	
Hepatic Flexure	6 (6.9%)	6 (3.1%)	
Transverse	2 (2.3%)	5 (2.6%)	
Splenic Flexure	1 (1.2%)	3 (1.5%)	
Descending	4 (4.6%)	9 (4.6%)	
Sigmoid	27 (31.0%)	47 (24.2%)	
Rectum	38 (43.7%)	90 (46.9%)	
T Stage, n (%)			0.434
T1	17 (19.5%)	21 (10.8%)	
T2	15 (17.2%)	37 (19.1%)	
T3	43 (49.4%)	115 (59.3%)	
T4	12 (13.8%)	21 (10.8%)	
N Stage, n (%)			0.361
N0	50 (57.5%)	102 (52.6%)	
N1	21 (24.1%)	44 (22.7%)	
N2	16 (18.4%)	48 (24.7%)	
Lympho-vascular Invasion, n (%)	25 (28.7%)	60 (30.9%)	0.688
Peri-neural invasion, n(%)	17 (19.5%)	33 (17.0%)	0.625

Group A* - Patients who had all synchronous polypectomy sites resected

*Group B^ - Patients who had synchronous polypectomy sites left* in situ

### Metachronous tumours

Two (0.7%) patients developed local recurrences while six (2.1%) patients developed metachronous colorectal tumours. The details of the six patients who developed metachronous lesions are illustrated in Table 3.

**Table 3:** Characteristics of patients who developed metachronous lesions

Patient	1	2	3	4	5	6
Initial tumour site	HF	Rectum	Caecum & AC	Rectum	DC & SC	AC
Histology/stage	AdenoCa T3N0M0	AdenoCa T3N2M0	AdenoCa T3N2M0	AdenoCa T3N0M0	AdenoCa T2N0M0	AdenoCa T3N2M1
Margins of initial resection	Clear Proximal - 17 cm Distal - 3 cm	Clear Proximal - 13 cm Distal - 2 cm	Clear Proximal - 5.5 cm Distal - 8.5 cm	Clear Proximal - 11.5 cm Distal - 2.5 cm	Clear Proximal - 2 cm Distal - 3.5 cm	Clear Proximal - 20 cm Distal - 6 cm
Surgery	Right Hemicolectomy	Low Anterior Resection	Right Hemicolectomy	Low Anterior Resection	Left Hemicolectomy	Right Hemicolectomy
Synchronous polypectomy numbers	1	4	2	2	1	1
Polypectomy site(s)	DC	a) SC b) SC c) DC d) DC	a) AC b) TC	a) Rectum b) DC	TC	SC
PS Proximal/Distal to tumour	Distal	Proximal	Distal	Proximal	Proximal	Distal
Polyp(s) morphology	Pedunculated	a) Pedunculated b) Pedunculated c) Sessile d) Sessile	a) Sessile b) Sessile	a)Pedunculated b) Sessile	Sessile	Pedunculated
Polyp histology	TA LGD	a) TA LGD b) TA HGD c) HP d) HP	a) HP b) HP	a) TA LGD b) TA LGD	TA LGD	TVA HGD
Size (mm)	15	a) 15 b) 12 c) 6 d) 7	a) 3 b) 3	a) Missing b) Missing	3	10
All PS included in resection	No (Group B)	No (Group B)	No (Group B)	Yes (Group A)	No (Group B)	No (Group B)
Site of metachronous tumour	DC	DC	TC	DC	HF	TC
Interval to development of metachronous lesion (months)	32	11	11	36	83	9
Metachronous lesion at colonic segment of previous synchronous PS?	Yes	Yes	Yes	Yes	No	No

*HF* Hepatic flexure, *AC* Ascending colon, *DC* Descending colon, *SC* Sigmoid colon, *AdenoCa* Adenocarcinoma, *TC* Transverse colon, *TA LGD* Tubulo-adenoma with low grade dysplasia, *TA HGD* Tubulo-adenoma with high grade dysplasia, *HP* Hyperplastic, *TVA HGD* Tubulo-villous adenoma with high grade dysplasia, *TVA LGD* Tubulo-villous adenoma with low grade dysplasi

Four of the six patients (Patients 1 to 4) with metachronous tumours developed lesions at the same segment of the residual colon where their previous synchronous polypectomy was performed. In the remaining 2 patients (Patients 5 & 6), one had a metachronous tumour at the hepatic flexure (initial polypectomy site in the transverse colon) while the other had a metachronous tumour in the transverse colon (initial polypectomy site in the sigmoid colon).

The rates of metachronous tumours occurring at the same colonic segments of the previous synchronous PS in groups A and B were 1.1% (1 out of 87) and 1.5% (3 out of 194), respectively (*p* = 0.795). Among these four patients, two had initial PS located proximal to the primary cancer (Patient 2 and 4) while the other two had PS located distal to the primary cancer (Patient 1 and 3).

## Discussion

This study is one of the first to analyse potential tumour implantation risks after a polypectomy in the setting of a newly diagnosed colorectal malignancy. The decision of concurrent endoscopic polypectomy in a malignant lesion is often variable. Often, endoscopists may remove small polyps during intubation, prior to reaching the cancer, to avoid missing these lesions during withdrawal. In other circumstances, when a significant polyp is diagnosed synchronously, but at a considerable distance from the cancer, the polyp may be removed to obtain a histology. This will aid the decision for surgery as a malignant histology of a large polyp will require a concurrent bowel resection of the site, or the surgeon may choose to leave a portion of the bowel alone if the histology is benign. This has an important bearing on various technical considerations as performing an anastomosis may be more difficult when bowel lengths are reduced. Furthermore, this decision has important implications for patients as the functional outcomes of patients can be affected adversely in an extensive bowel resection, which can result in copious diarrhoea after surgery.

Our study has demonstrated that the incidence of tumour seeding at the PS after synchronous polypectomy, if any, is very low. This is evident as the rates of metachronous tumour that developed at the same colonic region of previous synchronous polypectomies were similar in patients from groups A and B (1.1% vs 1.5%, *p* = 0.795). If the incidence of tumour implantation in PS was significant, one would expect those in group B to have a far higher incidence of metachronous tumour when compared to those in group A. This was not the case in our study and alludes that the risk of tumour implantation on PS is likely insignificant. The cited rates of a 5-year local recurrence after curative treatment for colorectal cancer range from 2 to 5% while that of metachronous lesions range from 3 to 10% [13–16]. The metachronous lesion rate of 2.1% in our study cohort, all of whom had synchronous polypectomy performed, lies within the lower limit of these figures. This further reiterates that risks of tumour implantation after synchronous polypectomy is likely to be negligible.

These findings are also consistent with the systematic review by Sheel et al. [9]. In this systematic review, the only identified literature that studied human subjects was an isolated case report [10]. In this case report, a patient was diagnosed with a long segment primary rectal tumour of 1.5 cm to 12 cm from the anal verge and had proximal colonic biopsies during the index colonoscopy. This was performed before the rectal cancer was biopsied. After neoadjuvant radiation treatment, the patient underwent abdominal-perineal resection and was noted to have an area of mucosal irregularity, 12 cm proximal to the tumour. This area was subjected to histopathology analysis, which revealed granulation tissue near the mucosal surface; however, malignant tumoural glandular structures were detected in the submucosa. This led the authors to allude that the findings may be related to tumour seeding. However, another possible explanation could also be a sub-mucosal extension or lymphatic or vascular invasion of the primary lesion, an alternative made even more plausible in view of the poorly differentiated histology of the primary malignant lesion.

The low risk of mucosal implantation of tumour cells has also been demonstrated in mammalian studies. Yu et al. performed a study assessing tumour implantation in rabbits and failed to demonstrate any cases of mucosal implantation in the study [17]. Tumour implantation appeared to predominantly occur in areas where the serosa of the bowel was breached, with the tumour in-growth from the serosa inwards. In another study, Broyn et al. assessed the risks of tumour implantation in the damaged colonic mucosa of rats and similarly concluded that damaged colonic mucosa is extremely resistant to tumour seeding [18].

Two factors ought to be considered in the mechanism of tumour implantation in the colonic mucosa: 1) the presentation of viable malignant cells and 2) the susceptibility of the mucosa to allow the proliferation of malignant cells. In addition to the intrinsic ability of the colonic mucosa to resist tumour implantation, the distribution pattern of exfoliated cancer cells may also explain the low incidence of mucosal implantation in clinical settings. Maeda et al. assessed the distribution of exfoliated colonic tumour cells and concluded that malignant cells were predominantly found within 5 cm from the primary tumour [19]. Within 5 cm proximal and distal to the primary tumour, exfoliated malignant cells were found in 25 to 90% of the specimens compared to in 5 to 15% at distances beyond 5 cm. The findings suggest that PS at the greatest risk of exposure to exfoliated malignant cells lie within 5 cm from the primary tumour [20]. 5 cm is the current recommended resection margin for colon cancer and thus the at-risk mucosa in these regions would usually have been resected in an oncological resection.

There are several limitations to our study.

Data on the location (colonic segment) of the polyps removed were based mainly on endoscopists’ descriptions, which are prone to an error rate of close to 20% [21]. In mitigation, this error rate is likely to be evenly balanced during the comparisons between groups A and B. There is also evidently treatment bias in this study as significant polyps or polyps with high risks of implantation due to the proximity of the tumours may have been removed surgically, thus not completely excluding this hypothesis. The use of metachronous tumors as a surrogate for PS implantation is also contentious and ideally, the metachronous cancers that were detected should be subjected to genomic analysis and compared to the initial malignant lesion to determine true malignant implantation. However, this would be costly and we did not have the necessary funding to pursue this verification.

It would also be interesting if the metachronous cancer rate of our study cohort could be compared with the other 3113 patients with colorectal cancer without synchronous polypectomy (Fig. 1). Unfortunately, we do not have the complete follow up data for these 3113 patients for comparison. In addition, with our study cohort demonstrating such a low metachronous cancer rate of 2.1%, it is highly unlikely that the patients without synchronous polypectomy would have a metachronous cancer rate that is significantly lower. Nonetheless, this study represents an invaluable addition to literature on the possible risks of tumour implantation after synchronous polypectomy in the setting of a newly diagnosed colorectal cancer. The incidence of mucosal implantation after synchronous polypectomy is so infrequent that it would be impractical to expect evidence in the form of randomized controlled trials to guide our clinical practice. A meta-analysis of published clinical studies represents the most practical alternative once a critical mass of studies is available.

## Conclusion

Malignant implantation after synchronous polypectomy in the setting of a newly diagnosed cancer remains unproven. Even if tumour implantation did occur, the incidence is likely low.

## Abbreviations

CEA: Carcinoembryonic antigen
PS: Polypectomy sites
